# Task-shifting and family planning continuation: contraceptive trajectories of women who received their method at a community-based event in Kinshasa, DRC

**DOI:** 10.1186/s12978-023-01571-6

**Published:** 2023-01-30

**Authors:** Julie H. Hernandez, Katherine H. LaNasa, Tesky Koba

**Affiliations:** 1 Department of International Health and Sustainable Development, Tulane University School of Public Health and Tropical Medicine, 1440 Canal St, Suite 2200, New Orleans, LA 70112 USA; 2 Department of Health Policy and Management, Tulane University School of Public Health and Tropical Medicine, 1440 Canal St, Suite 1900, New Orleans, LA 70112 USA; 3 Kinshasa School of Public Health, University of Kinshasa, Kinshasa, Democratic Republic of the Congo

## Abstract

While community-based interventions are a proven high-impact strategy to increase contraceptive uptake in low-income countries, their capacity to support women’s contraceptive choices (including continued use, switching and discontinuation) in the long run remains insufficiently discussed. This cohort study follows 883 women 3 and 6 months after they received a modern method during community campaigns organized in Kinshasa (D.R. Congo), to analyze their contraceptive trajectories and the factors associated with ever discontinuing contraceptive use in the first 6 months following a campaign. In the community-based distribution (CBD) model currently institutionalized in DRC, campaign clients are not provided with additional doses or support, besides baseline counseling, to (dis-)continue using the method they received, but must rely on Family Planning resources within the existing local health system. Almost a third (28.9%) of all women discontinued modern contraception during the study period, with much higher discontinuation rates for short-acting methods (38.7% for pills and up to 68.9% for DMPA-SC). Variables previously associated with high discontinuation (marital status, fertility intentions and side-effects) led to higher odds of “ever discontinuing”. However, these variables became non-significant when controlling for resupply issues. Women’s self-reported reasons for discontinuation confirmed the multivariate regression results. Detailed sub-analysis of resupply issues for pills, injectables and Cyclebeads pointed to the role of cost, unreliable campaign schedules and weak integration of community-based strategies into the formal health system. Extremely low rates of implants removal suggest similar access to FP services issues. The study highlights the need to identify CBD strategies best suited to support women’s choices and preferences towards successful contraceptive trajectories in fragile health systems.

**Supplementary Information:**

The online version contains supplementary material available at 10.1186/s12978-023-01571-6.

## Plain Language Summary

In countries with insufficient access to health facilities, such as the D. R. Congo, the provision of contraceptives through campaigns implemented in community settings is a commonly used strategy to increase the number of contraceptive users. However, the long-term trajectories of campaign clients are not often examined, and it is unclear whether these events can be an effective way to support women’s choices and preferences when it comes to using, switching or stopping a contraceptive method. Our study looks at the contraceptive trajectories of 883 women who obtained a method during a community event to see whether they were still using that a modern contraceptive method 3 and 6 months later. Almost a third of the women discontinued at some point in that period, with much higher discontinuation rates for monthly pills and injectable contraceptives. While some individual characteristics (marital status, desired number of children, experienced side-effects) influenced discontinuation risk, the most important factor was the woman’s ability to resupply her method. Implant removal was also much lower than expected and this suggested difficulties in accessing existing family planning services. Our findings indicate that campaigns are still too disconnected from existing family planning services to successfully support women’s contraceptive choices in the long run.

**Supplementary Information:**

The online version contains supplementary material available at 10.1186/s12978-023-01571-6.

## Background

Community-based provision of primary care services, including contraceptive distribution, has long been used as a high impact intervention to mitigate gaps in fragile health systems [1], particularly in Sub-Saharan Africa [2–4]. In the Democratic Republic of Congo (DRC), outreach programs were implemented as early as the 1980s, first in rural environments before being extended to urban centers, to increase contraceptive availability where health facilities were too distant and providers were insufficiently trained [5]. Three decades later, family planning (FP) indicators in the DRC remain weak with modern contraceptive prevalence (mCPR) in the capital city of Kinshasa only reaching 30.3% in 2021 and unmet need increasing to 17.6% for women married or living in union [6]. Women seeking FP services from health facilities continue to face strong barriers to access including costs, provider attitudes, and frequent stock outs [7, 8].

In response to these issues, new community-based programs have been piloted, refined and scaled-up in the DRC since it became a focus country for the FP2020 initiative [9]. Initially, several programs used community health workers (CHW) to individually provide FP services in their own neighborhood, but program evaluations indicated this strategy faced several issues previously noted for this model of community-based distribution in low-income countries [9, 10]. These include difficulties incentivizing the CHW to work alone, limited range of contraceptives authorized for distribution by CHWs with no medical background, frequent commodities stock-outs, weak collaboration with health facilities for resupply, referral systems and service statistics reporting and overall low levels of activities [1].

In parallel to these “prototypical” [10] CHW models however, the DRC also developed an innovative service delivery strategy using 3^rd^ and 4^th^ year Nursing School (NS) students as community-based distributors (CBD) to provide FP services during large community campaigns as part of their practicum training [11–13]. These campaigns take place in a community setting (marketplace, soccer field, community center yard) using a nearby health facility for referral and provision of additional methods. The events are organized jointly with local health zone officers to centralize contraceptive provision, resupply, and reporting activities. The student CBDs offer an extended range of methods, including DMPA-SC and Implanon NXT, the latter being one of the most commonly used methods in DRC (36.8% of married modern contraceptive users per PMA 2022 data [6]). Methods are provided for free or at a heavily discounted prices, and the student CBDs are trained to provide client-centered counseling. Programmatic evaluation of the NS model indicated the student CBDs were motivated by working in a group and highly valued the opportunity to practice their counseling and service provision skills during the campaigns [14]. Under the existing model, NS students participate in three to four campaigns per year in 93 health zones across the seven provinces where the model is currently implemented. As of 2022, the intervention covered a third of the country and scale-up has continued with additional donors and partners [14].

In other contexts with low contraceptive prevalence, community-based provision of contraceptive services has proven effective at capturing and supporting new and existing FP users, delivering high volumes of contraceptives, and has contributed to increasing mCPR [15, 16]. However, cross-sectional evaluations of these programs tend to focus on single client visits (particularly new users) and quantities of methods provided [17, 18], and do not always consider the contraceptive trajectories of their clients after they received their method, be it from an individual provider or during a community-based event. Yet, for FP programs to successfully support women’s contraceptive choices, they must not only contribute to contraceptive initiation but also foster long-term use of the woman’s preferred method(s), and enable her to switch and stop contraception based on her preferences and life decisions [19, 20]. As one of the key CBD models currently scaled up and institutionalized in the DRC, the nursing schools’ campaigns are effective at providing high volumes of contraceptives, but it is unclear whether they can adequately support the FP needs of their clients in the long term.

The calendar tool in the Demographic and Health Survey has contributed evidence for multiple cross-country analyses looking at (dis)continuation rates of contraceptive methods and associated factors. Analyses from DHS surveys conducted between 2010 and 2014 revealed that, on average, between 17.5 and 50.0% of women in Sub-Saharan Africa discontinued using modern contraception within 12 months, and 6.0–36.7% of women switched to another modern method within 3 months after discontinuation [21]. Socio-demographic characteristics such as younger age [22], marital and lower parity status [23, 24], and higher fertility wishes [25] have been found to be associated with higher odds of ever discontinuing method use, as well as experiencing side-effects [23, 26]. In addition, there is growing evidence that the quality of care and counseling received at the point of method of initiation is strongly associated with method continuation [27–29]. However, few studies have specifically included the role of the broader FP supply environment on clients’ contraceptive trajectories.

The main objective of this study is thus to observe the contraceptive trajectories of women who were provided a modern method and counselled on additional FP services available in their neighborhood during nursing school campaigns in Kinshasa, DRC. The analyses will further assess incidence of contraceptive interruption, and associated variables, among women who were still in need of a modern contraceptive method in the 6 months following a campaign event. Findings from this study will contribute to better understanding how, in the context of fragile FP environments, community-based campaigns, which are key to the DR Congo’s FP strategy [30], can act as an effective gateway for supporting women’s long-term contraceptive choices..

## Methods

A cohort of 1112 women were initially recruited during FP campaigns organized by nursing schools in four health zones of Kinshasa (April–May 2021). Women attending a campaign first received group counseling and a presentation of all contraceptive methods available. Then, they met one-on-one with student CBDs for individual counseling and method selection. At the community level, nursing students are authorized to provide condoms, oral pills, emergency contraception, and Standard Days Method (locally known as “Cyclebeads”), and to inject DMPA-SC or to insert the 3-year Implanon NXT. Women who wished to receive the 5-year Jadelle implant or an IUD were referred to a nearby health facility supporting the campaign. During the campaigns, contraceptives were provided for free or at a heavily discounted price, and there were no additional client fees. Counseling included information on the method’s efficacy, duration, and possible side-effects and how to address them. For DMPA-SC users, women were counseled following the DRC guidelines for reinjection, which include a 2-week grace period beyond 3 months before the method efficacy declines. For short-acting methods, clients were also told about existing resupply locations in their neighborhood, including health facilities and pharmacies. As the campaigns’ schedule tends to be irregular, clients were not given information on upcoming similar events. For long-acting methods, users were provided information on removal opportunities at local health facilities. After completing the counseling and method selection, women were then told about the possibility to participate in the study and, if interested, directed towards a data collector in a private area near the campaign location.

Data collectors were women with several years of experience in conducting surveys related to sexual and reproductive health (including PMA surveys), fluent in all local languages, and who had been specifically trained to collect information for this study by the research team in partnership with the Kinshasa School of Public Health. Training also included ethical considerations, particularly for recontacting and following-up with participants in their community. During the baseline survey, respondents who agreed to participate in follow-up surveys provided data collectors with two alternate ways of recontacting them. To account for the 2-week resupply grace period of DMPA-SC users, participants were recontacted at 14 weeks (August–September 2021) and 26 weeks (November–December 2021) after baseline (April–May 2021). For clarity in the analyses, the follow-up surveys are simply termed 3- and 6-months. During the follow-up rounds, each data collector attempted to recontact participants in their baseline pool twice. Respondents could elect to complete the follow-up survey over the phone or in-person at a location of their choosing. The response rate at 3-months was 89.3% and 90.0% at 6-months, which is comparable to other cohort studies conducted among FP users in Sub-Saharan Africa [31, 32]. Informed consent was obtained prior to each of the three survey rounds. Data for all surveys was collected on password-protected smartphones using the OpenDataKit (ODK) app. Field supervisors checked and validated all survey forms before submitting them to an encrypted cloud service for database aggregation.

### Data analysis

The analytic sample was restricted to a panel of 883 women who had selected a method provided at the community event and completed all three surveys. The main outcomes measured for this analysis included “ever discontinued modern contraception in the 6 months following the campaign”, as well as reasons given for discontinuation. Discontinuation is measured as “not using any modern contraceptive method at either the three- or 6-months follow-up interview”. Women who received a method during the baseline campaign but reported using a different one (switching) at 3 or 6 months are not considered discontinuers. Description of the cohort at baseline assessed socio-demographic characteristics and detailed FP history, including the method selected at baseline. We then analyzed (dis-)continuation and switching trends by method at 3 and 6 months and self-reported reasons for discontinuation. We then ran multivariate logistic regression models with random effects to predict the odds of discontinuation while still in need of contraception during the 6 months following initiation, using independent variables known to be associated with FP continuation, including demographic characteristics, experiencing side-effects, counseling received during the campaigns, and resupplying at least once during the study period.

## Results

### Cohort description and baseline data

The analytic sample for this study is restricted to women who selected a method provided at the community event and completed all three interviews. Out of 1112 women interviewed at baseline, 17 selected a method that needed a referral to a health facility (6 Depo-Provera injections and 11 Jadelle implants) and 212 were lost to follow-up, leaving a total panel of 883 women for the analysis. There were no significant differences in the distribution of four out of five baseline demographic characteristics between women who completed all interviews and those lost to follow-up. There was, however, a significant difference in education level, where a higher percentage of women lost to follow-up (17.92%) had no education compared to women who remained in the study (9.17%). In addition, the distribution of methods selected at baseline was significantly different among women lost-to-follow up compared to those who completed all surveys. The majority (51.9%) of women lost-to-follow up received an Implant NXT at baseline compared to 39.2% of women who completed all interviews, while no method dominated over half the method mix selected among women who completed all survey (Additional file 1: Table S1).

Out of the 883 women in the analytic sample, nearly half (45.1%) were youth under 24 years old, 50.9% were married or living in union, and most had completed primary education (59.6%) or higher (31.3%). The majority of women (86.2%) had at least one child, with more than a third (36.2%) having more than three children at the time they visited the campaign. When asked about fertility intentions, most women (78.8%) wished to wait more than 2 years until they became pregnant again, but only 13.5% declared not wanting any more children. Table 1 details the demographic characteristics of the cohort.

**Table 1:** Baseline characteristics of women who completed all three interviews

Baseline demographics (%)	n	%
*Age (years)*		
15–24	398	45.1
25–34	346	39.2
35–49	139	15.7
*Marital status*		
Not married	434	49.2
Married/living in union	449	50.9
*Education level attained*		
None	81	9.2
Primary	526	59.6
Secondary or higher	276	31.3
*Parity*		
0	122	13.8
1–2	441	49.9
3–4	212	24.0
5 +	108	12.2
*Time preferred until next child*		
< 1 year	11	1.3
1–2 years	57	6.5
More than 2 years	696	78.8
No more children	119	13.5
*Total*	883	100.0

Table 2 outlines the mix of methods selected at baseline and clients’ previous experience using FP. Out of the five contraceptive methods available at the community-level (excluding condoms, which are provided to all campaign clients without distinction for STI prevention purposes), a majority of women (38.4%) selected the 3-year implant (Implanon NXT), followed by DMPA-SC (26.6%), oral pills (19.0%), CycleBeads (15.2%) and Emergency Contraception (0.8%). When asked about their FP history, three quarters of women (74.9%) had used any FP method (traditional or modern) prior to their visit to the campaign, while 59.2% had used a modern method previously. CycleBeads clients were the least experienced users with 41.8% reporting they were new FP users. A third (31.4%) of the clients who had used modern contraception previously reported prior experience with the specific method they selected during the campaign, with EC (50.0%), oral pills (51.7%) and DMPA-SC (36.6%) clients being the most experienced users. Given the small sample of women who selected emergency contraception at baseline (n = 7), these women were excluded from all further analyses.

**Table 2:** Method selection and FP history at baseline (%)

	All methods	Method selected at baseline				
		EC	Cyclebeads	Orals Pills	DMPA-SC	Implanon NXT
	(n = 883)	(n = 7)	(n = 134)	(n = 168)	(n = 235)	(n = 339)
*Method selected*	100.00	0.8	15.2	19.0	26.6	38.4
*History with family planning*						
New to FP	25.0	0.0	41.8	28.0	12.3	26.3
Used traditional method previously	15.7	14.3	21.6	20.2	8.5	16.2
Used a modern method previously	59.2	85.7	36.6	51.8	79.2	57.5
*History with selected method (among women who used a modern method previously*)	(n = 523)	(n = 6)	(n = 49)	(n = 87)	(n = 186)	(n = 195)
New method user	68.6	50.0	75.5	48.3	63.4	81.5
Used method previously	31.4	50.0	24.5	51.7	36.6	18.5

Looking at clients’ characteristics associated with specific methods, we found that having a lower level of education, being married, higher parity and wanting to wait more than 2 years to have another child were positively associated with selecting a long-acting method (chi-squared test—p-value < 0.05. See Additional file 2: Table S2).

### Contraceptive trajectories at 3 and 6 months

**Fig. 1:**
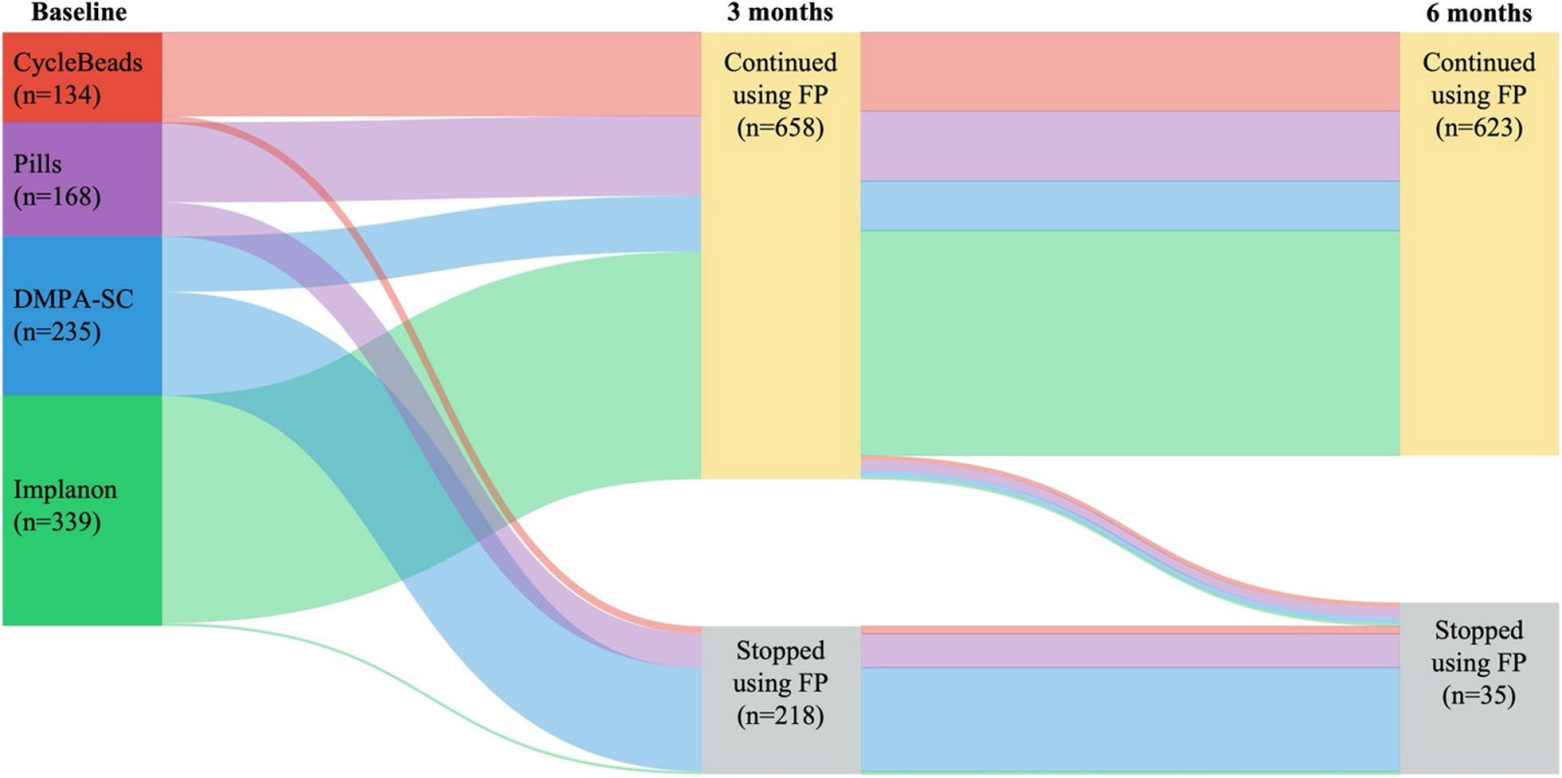
Trends in contraceptive (dis-)continuation patterns for the cohort

Out of 876 women in the analytic cohort, 623 (71.1%) used modern contraception continuously during the first 6 months following the campaign; this includes 75 women who at some point switched from their baseline method to another contraceptive. Out of the 253 women who discontinued contraception during the study period, most (86.2%) did so during the first 3 months of use. Figure 1 shows the cohort trajectory over the study period.

The overall discontinuation rate, however, masks important differences in method trajectories, as showed in Table 3.

**Table 3:** (Dis-)continuation patterns at 3 and 6 months by method

	All methods	CycleBeads	Oral Pills	DMPA-SC	Implanon NXT
*Trajectory*	(n = 876)	(n = 134)	(n = 168)	(n = 228)	(n = 346)
Used continuously	71.1	86.6	61.3	31.1	97.6
Ever discontinued	28.9	13.4	38.7	68.9	2.4
*Discontinuation time point*	(n = 253)	(n = 18)	(n = 65)	(n = 162)	(n = 8)
Three months	*86.2*	*61.1*	*76.9*	*94.4*	*50.0*
Six months	*13.8*	*38.9*	*23.1*	*5.6*	*50.0*

EC users were excluded due to the small sample size (n = 7)

The least discontinued method was Implanon NXT (2.4% of clients ever discontinued the method in the 6 months following the campaign), followed by CycleBeads (13.4% ever discontinued). DMPA-SC was by far the most discontinued method; at 3-months (14 weeks), the majority (65.1%) of women who had selected the method at baseline had not received their second injection, and by 6-months (26 weeks), 68.9% reported missing at least one injection during the study period. Similarly, 38.7% of monthly pill clients also discontinued the method and were no longer using modern contraception at some point in the first 6 months.

### Determinants of discontinuation for campaign clients

Table 4 details the main reason given for method discontinuation for all clients who ever stopped using modern contraception in the first 6 months after the campaign. Nearly all women who stopped using a method were still in need of contraception, only 2.4% and 1.6% of discontinuers wanted to or did become pregnant during the study period. There were four recorded method failures on the total sample. Among women who discontinued while still in need, resupply issues were the most commonly reported reasons (54.2% overall, including CycleBeads users who lost the device), followed by health and fertility concerns (19.4%) and infrequent sex (13.8%). Partner or family opposition represented only 2.8% of reasons stated for discontinuation.

**Table 4:** Self-reported reasons for method discontinuation

Main reason reported for discontinuation (%)	Total	CycleBeads	Pills	DMPA-SC	Implanon NXT
	(n = 253)	(n = 18)	(n = 65)	(n = 162)	(n = 8)
Resupply issues	54.2	66.7	40.0	61.1	0.0
Health and fertility concerns	19.4	0.0	18.5	18.5	87.5
Infrequent sex	13.8	11.1	23.1	11.1	0.0
Opposition from partner or family	2.8	0.0	6.2	1.9	0.0
Wanted to become pregnant	2.4	0.0	5.2	1.2	0.0
Method failure	1.6	0.0	1.5	1.9	0.0
Other reason	5.1	11.1	4.6	4.3	12.5
No response	0.8	11.1	0.0	0.0	0.0

EC users were excluded due to the small sample size (n = 7)

Resupply issues affected clients across socio-demographic profiles and regression models yielded no significant associations between age, education, marital or parity status, fertility intention and FP history and the likelihood to experience resupply issues (see Additional file 3: Table S3).

The logistic regression results are presented in Table 5. Model 1 reports the association between odds of ever discontinuing and experience of side effects while using the method, baseline counseling experience, history of using FP, and sociodemographic variables among all clients in the cohort. In this model, women who were married/living in union and wanted to wait two or more years until their next child were significantly less likely to discontinue (OR = 0.36 p = 0.006; OR = 0.11 p = 0.026, respectively). Women who did not obtain their preferred method at baseline were significantly less likely to discontinue (OR = 0.27 p = 0.008). This association appears to be driven in part by Implanon NXT users who reported a higher proportion of clients who did not receive their preferred method at baseline (28.0%) and who also had a much lower discontinuation rate (2.4%) compared to other methods. Indeed, in subsequent models which exclude Implanon NXT users, this association becomes nonsignificant. Finally, women who had ever used modern contraception previously were significantly more likely to discontinue (OR = 2.32 p = 0.025), however, this association is reversed in subsequent models after controlling for resupply knowledge and experience. Model 2 is restricted to clients who selected short-acting methods at baseline and includes where women were told to resupply their method during baseline counseling. In this model, women who were married/living in union and those who had used the same method previously were significantly less likely to discontinue (OR = 0.31 p = 0.001; OR = 0.46 p = 0.046, respectively). Women who reported experiencing side effects had 5.7 higher odds of discontinuing compared to those who did not experience side effects (p < 0.001). Women who were told to resupply at a community location or at a health facility were also significantly more likely to discontinue compared to women who reported they were not told where to resupply (OR = 3.43 p = 0.003; OR = 3.66 p < 0.001, respectively). Finally, Model 3 is further restricted to women who selected a short-acting method that requires routine resupply (pills and DMPA-SC) and includes an independent variable for whether the woman resupplied the method at least once during the study period. In this model, women who were told to resupply at a health facility were significantly less likely to discontinue compared to those who reported they were not told where to resupply (OR = 0.49 p = 0.033). Similarly, women who resupplied their method at least once were significantly less likely to discontinue compared to those who never resupplied (OR = 0.01 p < 0.001).

**Table 5:** Odds of discontinuation during the 6-month study period among women in need of modern contraception

Independent variables	Model 1		Model 2		Model 3	
	OR	p-value	OR	p-value	OR	p-value
*Experience with method*						
Experienced side effects (after baseline)	1.70	0.072	5.69	< 0.001	1.60	0.128
Was able to resupply method at least once	–	–	–	–	0.01	< 0.001
*Baseline counseling experience*						
Intended to continue using FP at baseline	0.27	0.273	0.24	0.192	0.87	0.882
Did not obtain preferred method at baseline	0.27	0.008	1.19	0.738	1.15	0.753
Was told where to resupply (ref: No)						
Yes, community location	–	–	3.43	0.003	0.58	0.157
Yes, health facility	–	–	3.66	< 0.001	0.49	0.033
Yes, pharmacy	–	–	0.75	0.651	0.41	0.103
Yes, other	–	–	0.47	0.717	0.56	0.749
*History of FP use*						
Ever use modern contraception	2.32	0.025	1.37	0.381	0.53	0.054
Ever used that method previously	0.95	0.917	0.46	0.046	0.56	0.075
*Sociodemographics*						
Age group (ref: 15–24)						
25–34	0.98	0.960	0.67	0.327	0.55	0.089
35–49	1.57	0.474	0.75	0.623	0.76	0.596
Education level (ref: none)						
Primary	1.46	0.490	0.82	0.731	0.99	0.980
Secondary or higher	1.51	0.489	0.65	0.477	1.37	0.566
Married/living in union	0.36	0.006	0.31	0.001	0.59	0.091
Number of living children	0.92	0.578	1.21	0.184	1.14	0.293
Desired time until next child (ref: < 1 year)						
1–2 year	1.70	0.079	0.31	0.196	0.30	0.304
2+ years	0.11	0.026	0.27	0.143	0.33	0.332
No more children	0.13	0.064	0.25	0.175	0.28	0.312
*Observations*	869		530		388	

The sample for model 1 consists of all clients, the sample for model 2 consists of women who selected a short-acting method at baseline, and the sample for model 3 consists of women who selected a short-acting method at baseline which requires routine resupply (oral pills, DMPA-SC). EC users have been excluded from all models due to the small sample size (n = 7)

### Detailing resupply issues

Table 6 presents descriptive statistics for women who discontinued using modern contraception at any point in the first 6 months after receiving their method and specifically cited resupply issues as their main reason for discontinuation (n = 157).

**Table 6:** Experience of women who discontinued due to resupply issues

	3 months		6 months		Ever discontinued	
	n	%	n	%	n	%
*Resupply issue*	99	100.0	58	100.0	157	100.0
Cost	35	35.4	30	51.7	65	41.4
Did not know where to go	26	26.3	12	20.7	38	24.2
Waiting for next campaign	19	19.2	7	12.1	26	16.6
No time	9	9.1	2	3.5	11	7.0
Stockout	2	2.0	2	3.5	4	2.5
Too far	0	0.0	1	1.7	1	0.6
Other	8	8.08	4	6.9	12	7.6
*Were you told where to resupply?*	99	100.0	58	100.0	157	100.0
Yes	70	70.7	45	77.6	115	73.3
No	29	29.3	13	22.4	42	25.8
*Where were you told to resupply?*	70	100.0	45	100.0	115	100.0
Health facility	40	57.1	31	68.9	71	61.7
Community event/worker	24	34.3	12	26.7	36	31.3
Pharmacy	6	8.6	2	4.4	8	6.9
Other	0	0.0	0	0.0	0	0.0
*Number of resupply attempts*	99	100.0	58	100.0	157	100.0
None	82	82.8	43	74.1	125	79.6
1	13	13.1	10	17.2	23	14.7
2	4	4.0	2	3.5	6	3.9
More than twice	0	0	3	5.2	3	1.9
*Where did you attempt to resupply?*	17	100.0	15	100.0	32	100.0
Health Center	12	70.6	10	66.7	22	68.8
Pharmacy	5	29.4	5	33.3	10	31.3
CBD or campaigns/Lelo PF	0	0.0	0	0.0	0	0.0
Other	0	0.0	0	0.0	0	0.0

Overall, the results highlight that the vast majority (79.6%) of clients who discontinued due to resupply issues never visited a resupply location. Cost issues were the most frequently mentioned reason for not being able to resupply (41.4%), followed by not knowing where to go (24.2%). “Waiting for the next campaign” (16.6%) was not an option initially offered in the follow-up surveys but appears nonetheless as the third most commonly reported reason (given under “Other, specify”) and was thus recoded as an independent variable in the analysis. When asked about the counseling they received at baseline, three quarters (73.3%) of this subsample recalled being told where to go to resupply their short-acting method, with most of them reporting they were told to go to a health center (61.7%), followed by visiting a community event / contacting a community worker (31.3%), and going to a pharmacy (6.9%). The remaining women in this subsample reported attempting to resupply once (14.7%), twice (3.8%) and up to three times. When asked about where they attempted to resupply, clients only mentioned health centers (68.8%) and pharmacies (31.3%).

## Discussion

The nursing school campaign model is one of the cornerstones of the DRC’s community-based contraceptive provision strategy and has been institutionalized and scaled-up throughout the country. While the model has demonstrated its value regarding the number of clients and volume of contraceptives it can efficiently offer compared to other CBD programs, results from this study highlight issues regarding the campaigns’ capacity to adequately support women’s contraceptive choices in the long run. Nearly a third of women who received a modern FP method during a community-based campaign in Kinshasa discontinued using contraception at some point during the 6 months following the event, and as nearly all clients declared not wanting another child in the near future, this trajectory placed them at risk for unintended pregnancies.

Most discontinuers had received methods requiring timely (pills and injectables) or occasional (CycleBeads) resupply and this research returned discontinuation rates comparable to those recorded in other Sub-Saharan countries (e.g., 30% discontinuation at 12 months for a cohort of oral pill users in Southwest Ethiopia, see [33]). Determinants of method discontinuation were also similar to other studies: a prospective cohort study conducted in Kenya indicated that two-thirds of short-acting method clients stopped using that method 18 months after initiation and that challenges related to resupplying the method accounted for half of the discontinuations [34]. In our own cohort, resupply issues were the strongest determinant of discontinuation for short-acting methods, far beyond clients’ marital status, fertility intentions or experience of side-effects. In addition, our results signal that more experienced users might have better outcomes in terms of continuity (“having used that method before” was associated with significantly lower risks of discontinuing in the first 6 months among short-acting users), possibly because they already knew how to overcome these resupply challenges. While the existing literature tends to include both side-effects and resupply issues under “method-related problems” and shows that together they contribute for more than half of contraceptive discontinuation incidence [35], this study indicates a need to consider them separately since, for short-acting methods, the influence of side-effects on odds of ever discontinuing becomes non-significant when controlling for “resupplied at least once.”

The trajectories of Implanon NXT clients also points to issues in health system integration and access for campaign clients. The implant was the most commonly selected method in this study and yet the percentage of users who had their implants removed during the first 6 months (2.4%) was lower than the figure estimated in a similar study conducted in Kinshasa which showed a removal rate of 5.5% over the same time period (and up to 20.0% in the first 2 years after insertion [36]) and the average 12-month discontinuation rate of implants (13.6%) in other African contexts [21]. Considering the dearth of trained providers and cost of implant removal in DRC, additional research is needed to determine whether this relatively high continuation rate is the result of women’s choices or if it is artificially driven by barriers to accessing removal services.

This study highlights several issues for the current community-based model implemented in DRC to act as a gateway sustainably supporting women’s contraceptive choices, including continued use, switching or discontinuation of the method selected during the campaigns. Prior research evaluating the feasibility and acceptability of providing contraceptives at community-based events showed they were appreciated by women for their accessibility and the low cost of FP services offered [13, 37]. Indeed, considering the data presented in this paper, we hypothesize that the “waiting for the next campaign” response spontaneously given to the “why did you not resupply your method” question might be code for “waiting for contraception to be free / discounted”, which would be consistent with the notion of “cost” as the most frequently given reason for discontinuing short-acting methods. However, since most community-based provision of FP activities in Kinshasa are still largely dependent on donors or implementing partners, their schedules tend to be unreliable and limited by the agendas of international FP projects, thus making it difficult for clients to plan to resupply via these delivery points.

In addition, this study indicates issues in the counseling provided by student CBDs. A quarter of the women stated they had not been told where to go to resupply, and about a third reported the CBD recommended the client come back to see them for resupply when the CBD’s capacity to do so is subject to the same scheduling and supply chain constraints as those mentioned for the campaigns themselves. On the other hand, women who were referred to health facilities (which was both the most recommended and attempted resupply point), were effectively sent back to face the same barriers to FP access that community-based strategies were initially designed to address (i.e., cost issues, registration fees, untrained or unwelcoming staff, and frequent stock outs of short-acting methods). From this perspective, it is concerning that nearly four out of five women who declared having resupply issues did not attempt to find their next injection or their next box of oral pills once. The expectation of unsurmountable difficulties in accessing services is a well-known obstacle to an array of healthcare seeking behaviors [38, 39]. And, while participants in our study might have been taking advantage of the contraceptive services provided during the campaigns, the events do not appear to be adequate bridges to support women into continuing to seek these services within the existing health system.

This study is subject to several limitations. The significant difference in education levels for women lost-to-follow-up (LTFU) makes it difficult to apply our findings to women with no education, who may be facing steeper cost issues and other barriers to accessing FP services than the baseline clients included in our analytic sample. Considering the significantly higher proportion of Implanon NXT users among those LTFU, and the fact that women with lower levels of education were also more likely to select Implanon NXT at baseline, resupply issues might however be less of a factor in LTFU trajectories. This trend, however, limits the study’s capacity to adequately measure Implanon NXT continuation and removal attempts. In addition, there may be some endogeneity issues introduced at baseline if clients who selected a long-acting method did so because they already assumed they would face difficulties in resupplying a short-acting method. This would add to the long-term quality of care issues regarding CBD strategies if clients select methods based not on their own needs and preferences but on their expectation of encountering barriers in the existing contraceptive provision landscape. Finally, additional research may be needed to assess whether the issues highlighted in this study are specific to the “campaign-style” CBD model implemented in DRC, or if they affect other programs involving community-health workers operating in fragile environments.

## Conclusion

In order to successfully support women’s contraceptive choices (including continued use, but also method switching and discontinuation), it is essential that community-based contraceptive provision strategies provide information on additional, appropriate and cost-effective service delivery points for women facing barriers to access. For pills, injectables and CycleBeads in particular, this study showed resupply issues to be the main determinant of discontinuation in the months following community campaigns, with the majority of discontinuers still in need of contraception. Regular scheduling of community activities, adequate counseling messages, and stronger integration of community services with social marketing strategies to leverage the private sector are some of the opportunities emerging from this research that may be applied to other community-based projects implemented in low-income countries. Additionally, future research should strive to identify community-based strategies best suited to sustainably support women’s contraceptive choices and trajectories in fragile health systems.

## Supplementary Information

**Additional file 1: Table S1.** Distribution of baseline demographic characteristics among women who completed all interviews compared to women who were lost to follow-up.

**Additional file 2: Table S2.** Client demographic profile by method type selected at baseline.

**Additional file 3: Table S3.** Sociodemographic profile of women who reported resupply issues and health concerns as reasons for discontinuing use of contraception.

## Data Availability

All datasets for this study are protected under IRB regulations. Anonymized datasets are available upon motivated written request from the corresponding author for this study.

## Abbreviations

CBD: Community-based distributors
DMPA-SC: Subcutaneous (SC) depot medroxyprogesterone acetate
DRC: Democratic Republic of the Congo
EC: Emergency contraception
FP: Family planning
IUD: Intra-uterine device
LTFU: Lost to follow-up
mCPR: Modern contraceptive prevalence rate
ODK: Open data kit
