# Supplementary material for: Task-shifting and family planning continuation: contraceptive trajectories of women who received their method at a community-based event in Kinshasa, DRC
Source: Reprod Health. 2023 Jan 30;20:24. doi: 10.1186/s12978-023-01571-6 (PMC9887934; doi:10.1186/s12978-023-01571-6)
Supplement: Supplementary file 1 — Additional file 1: Table S1. Distribution of baseline demographic characteristics among women who completed all interviews compared to women who were lost to follow-up. [file 12978_2023_1571_MOESM1_ESM.docx]

Table S1. Distribution of baseline demographic characteristics among women who completed all interviews compared to women who were lost to follow-up.

| **Baseline demographics** | **Completed all interviews** | **Lost to follow-up** | **p-value** |
| --- | --- | --- | --- |
|  | (n=883) | (n=212) |  |
| Mean age | 26.7 | 26.0 | 0.171 |
| Mean number of children | 2.3 | 2.3 | 0.898 |
| Married/living in union (%) | 50.9 | 56.1 | 0.167 |
| Education level attained |  |  | **<0.001** |
| None | 9.2 | 17.9 |  |
| Primary | 59.6 | 59.0 |  |
| Secondary or higher | 31.3 | 23.1 |  |
| Time preferred until next child (%) |  |  | 0.437 |
| <1 year | 1.3 | 1.9 |  |
| 1-2 years | 6.5 | 4.3 |  |
| 2+ years | 78.8 | 77.8 |  |
| No more children | 13.5 | 16.0 |  |
| Method selected at baseline |  |  | **0.006** |
| EC | 0.8 | 0.9 |  |
| CycleBeads | 15.2 | 8.0 |  |
| Oral pills | 19.0 | 16.0 |  |
| DMPA-SC | 25.8 | 23.1 |  |
| Implanon NXT | 39.2 | 51.9 |  |
