# Supplementary material for: Task-shifting and family planning continuation: contraceptive trajectories of women who received their method at a community-based event in Kinshasa, DRC
Source: Reprod Health. 2023 Jan 30;20:24. doi: 10.1186/s12978-023-01571-6 (PMC9887934; doi:10.1186/s12978-023-01571-6)
Supplement: Supplementary file 2 — Additional file 2: Table S2. Client demographic profile by method type selected at baseline. [file 12978_2023_1571_MOESM2_ESM.docx]

*Table S2. Client demographic profile by method type selected at baseline*

| **Demographics (%)** | **n** | **Short-acting** | **Long-acting** | **p-value** |
| --- | --- | --- | --- | --- |
| Age (years) |  |  |  |  |
| 15-24 | 398 | 65.3 | 34.7 | 0.114 |
| 25-34 | 346 | 58.1 | 41.9 |  |
| 35-49 | 139 | 59.7 | 40.3 |  |
| Marital status |  |  |  |  |
| Not married | 434 | 66.8 | 33.2 | **0.002** |
| Married/living in union | 449 | 56.6 | 43.4 |  |
| Education level attained |  |  |  |  |
| None | 81 | 46.9 | 53.1 | **<0.001** |
| Primary | 526 | 59.3 | 40.7 |  |
| Secondary or higher | 276 | 70.3 | 29.7 |  |
| Parity |  |  |  |  |
| 0 | 122 | 91.8 | 8.2 | **<0.001** |
| 1-2 | 441 | 60.5 | 39.5 |  |
| 3-4 | 212 | 51.9 | 48.1 |  |
| 5+ | 108 | 50.9 | 49.1 |  |
| Time preferred until next child |  |  |  |  |
| <1 year | 11 | 90.9 | 9.1 | **<0.001** |
| 1-2 years | 57 | 91.2 | 8.8 |  |
| More than 2 years | 696 | 60.9 | 39.1 |  |
| No more children | 119 | 48.7 | 51.3 |  |
| Contraceptive history |  |  |  |  |
| New user | 221 | 59.7 | 40.3 | 0.507 |
| Experienced user | 662 | 62.2 | 37.8 |  |
