# Supplementary material for: Task-shifting and family planning continuation: contraceptive trajectories of women who received their method at a community-based event in Kinshasa, DRC
Source: Reprod Health. 2023 Jan 30;20:24. doi: 10.1186/s12978-023-01571-6 (PMC9887934; doi:10.1186/s12978-023-01571-6)
Supplement: Supplementary file 3 — Additional file 3: Table S3. Sociodemographic profile of women who reported resupply issues and health concerns as reasons for discontinuing use of contraception. [file 12978_2023_1571_MOESM3_ESM.docx]

*Table S3. Sociodemographic profile of women who reported resupply issues and health concerns as reasons for discontinuing use of contraception*

| **Baseline demographics (%)** | **n** | **Resupply Issue** | | **Health Concerns** | |
| --- | --- | --- | --- | --- | --- |
|  |  | **%** | **p-value** | **%** | **p-value** |
| Age (years) |  |  | 0.118 |  | 0.059 |
| 15-24 | 176 | 52.3 |  | 17.6 |  |
| 25-34 | 142 | 47.2 |  | 26.8 |  |
| 35-49 | 62 | 62.9 |  | 14.5 |  |
| Marital status |  |  | 0.893 |  | 0.251 |
| Not married | 231 | 52.4 |  | 18.6 |  |
| Married/living in union | 149 | 51.7 |  | 23.5 |  |
| Education level attained |  |  | 0.189 |  | 0.501 |
| None | 26 | 69.2 |  | 11.5 |  |
| Primary | 232 | 51.3 |  | 21.1 |  |
| Secondary or higher | 122 | 50.0 |  | 21.3 |  |
| Parity |  |  | 0.193 |  | 0.718 |
| 0 | 59 | 42.4 |  | 22.0 |  |
| 1-2 | 209 | 51.7 |  | 18.7 |  |
| 3-4 | 77 | 61.0 |  | 24.7 |  |
| 5+ | 35 | 51.4 |  | 20.00 |  |
| Time preferred until next child |  |  | 0.428 |  | 0.530 |
| <1 year | 14 | 35.7 |  | 21.4 |  |
| 1-2 years | 46 | 45.7 |  | 28.3 |  |
| More than 2 years | 283 | 53.4 |  | 19.8 |  |
| No more children | 37 | 56.8 |  | 16.2 |  |
| History with family planning |  |  | 0.463 |  | 0.148 |
| New FP user | 92 | 55.4 |  | 15.2 |  |
| Used any FP previously | 288 | 51.0 |  | 22.2 |  |
| History with selected method |  |  | 0.768 |  | 0.946 |
| New method user | 301 | 52.5 |  | 20.6 |  |
| Used method previously | 79 | 50.6 |  | 20.3 |  |
